# No impact of story context and avatar power on performance in a stop-signal game

**DOI:** 10.1016/j.heliyon.2024.e41039

**Published:** 2024-12-11

**Authors:** Linus Held, Johannes Pannermayr, Alina Kaufmann, Marouscha Scheffer, Paola Flores, Martin Dechant, Maximilian A. Friehs

**Affiliations:** a University of Twente, Netherlands; b University College London, United Kingdom; c University College Dublin, Ireland; d Max-Planck Institute for Human Cognitive and Brain Science, Germany

**Keywords:** Gamification, Stop-signal game, Inhibition, Motivation

## Abstract

This study investigates the impact of gamification on response inhibition in a Stop-Signal Task (SST) and examines participants' gamification experience. The findings reveal that, after accounting for approach- and avoidance-motivation as well as impulsiveness, higher immersion is associated with impaired response inhibition. This effect could be attributed to a substantial decline in immersion between the first and second SST sessions. Despite intrinsic motivation and avatar identification not significantly predicting performance, both factors exhibited a decline across sessions, suggesting an overall diminished gaming experience in the second session. Alternatively, motivational variables as immersion and avatar identification might be detrimental to response inhibition, by shifting attention away from relevant task elements. Contrary to expectations, approach and avoidance narratives did not influence outcome variables or participant experience, while different avatars led to altered avatar identification, particularly favouring strong avatars. The declining motivation over time might stem from a lack of tangible goals within the gamified task, where narrative elements alone failed to induce sufficient goal-oriented motivation. These findings underscore the nuanced interplay between gamification elements, task complexity, and participants' expectations, emphasizing the need for carefully tailored gamification strategies in experimental designs.

## 1 Introduction

In cognitive psychology there is generally a high need for experimental control, removing as many confounding factors as possible in order to gain reliable and valid results that are influenced only by the experimental manipulations. Thus, when probing processes like response inhibition, it is vital to rule out confounding factors that could pose a threat to its' measurement. Nevertheless, current cognitive research emphasises the role of gamification to sustain motivation in more realistic tasks, as the measurement of cognitive processes usually requires prolonged sustained attention. For this purpose, light-touch gamification is frequently used to sustain engagement with the task over time while keeping the experimental control as high as possible.^1^ Light-touch gamification in the present context describes the process of implementing unobtrusive gamification elements to a task, to keep the motivation as well as experimental control as high as possible. Thus, the game-like adaptations to the task need to be unobtrusive. Meaningful gamification can raise the intrinsic motivation of participants and can thereby foster long-term engagement with the task, thus enhance the quality of the measured data [3,4]. Nicholson [4] further argues that for the creation of meaningful engagement with a task, narratives can be used in gamification. However, it remains partially unclear how specific design or narrative choices influence motivation [5,6]. Hence, for cognitive psychology especially it is important to know what gamification elements influence performance and experience. While cognitive psychologists' aim is to keep experimental control high, game designers however may want to increase enjoyment. Thus, for effective gamification for a cognitive psychology application is to be as unobtrusive and as non-invasive as possible, while still improving motivation. Put differently, to create a light-touch gamification. But the questions remain: how much is too much, and how little is too little? The aim of this study is to investigate the effect of light-touch gamification and especially the effectiveness of narratives and different avatars in the Stop-Signal Game [[7], [8], [9]].

### 1.1 Gamification

Gamification is the process of making a task more engaging by adding game design elements to a task. For example, the gamified version of the SST is called Stop-Signal Game (SSG), which was validated in a study that highlighted the benefits of gamification, such as increased participant enjoyability of the task without compromising internal validity or enhanced ecological validity, as compared to the original SST [7,10]. Further, the SSG has been shown to be adaptable to incorporate different mechanics such as avatar customization [11] or incorporating food-specific stimuli [1].

Given the absence of explicit guidelines regarding the appropriate implementation of game elements to enhance participant motivation for cognitive performance tasks, a range of diverse approaches to gamification have emerged. Among other design choices, these gamification approaches vary in their inclusion of narratives and avatars to establish gamification strength. Narrative approaches for example focus on ways to foster the feeling of transportation to the narrative and potentially even identification with the characters in it [[12], [13], [14]]. Furthermore, framing a task as a game alone has shown to increase the enjoyability of a task [15]. Hence, the use of narratives in gamified tasks can be a good way to elicit changes in participants’ perception of a task, while keeping the experimental control as high as possible.

Further, many games feature the possibility to customize their own avatar in the game. Previous research into avatar identification through customization demonstrates that this can foster motivation, reduce experimental attrition, increase enjoyment, and improve the efficacy of cognitive training [2,11,[16], [17], [18]]. Avatar customization gives the player control, fosters a sense of autonomy, and makes the player feel as if they have more impact on the task [19]. However, avatar identification can be achieved not only by active engagement with the own digital representation, but merely the notion that this avatar represents the player can be sufficient [11,[20], [21], [22]]. Thus, in sum, the identification with the own avatar in game can be crucial for the players’ motivation and performance. In order to increase the experimental control, however, it might not be needed to customize avatars entirely but merely to present a relevant desirable aspect of the avatar with which the player can identify, such as avatar strength [23].

However, while gamification can certainly enhance engagement and motivation in cognitive tasks, its application is not without its complexities. Incorporating light-touch gamification may indeed provide heightened experimental control, ensuring participants' attention and compliance. On the flipside, this approach can also inadvertently fall short of its intended impact and even infringe upon participants' expectations, which then in turn leads to disappointments on the participants’ side and a reduced motivation [[24], [25], [26]].

### 1.2 The current study

In the current study we investigate the impact of light-touch gamification elements on cognitive task performance and motivation. We further investigate personality factors (i.e., impulsivity & approach-avoidance motivation) related to the game-design choices and the cognitive task in question. Impulsive individuals commonly exhibit a preference for risk-taking, a deficiency in planning and inhibitory control, and an inclination for immediate small rewards over delayed larger ones, frequently resulting in undesirable outcomes, such as impaired response inhibition [27]. The behavioural activation system (BAS) is activated by positive stimuli, including the reward itself, non-punishment, goal pursuit, as well as novel and exciting stimuli [28,29]. The behavioural inhibition system (BIS), on the other hand, is activated by a risk of punishment, loss of reward, and uncertainty [29]. Depending on current environmental cues, either one of these systems will be activated and can influence outcomes in the SSG [30]. For this reason, we included a personality measurement of impulsiveness and BIS/BAS activation to control for individual differences. Next, we adapted the original SSG [7] to include two distinctive character conditions, namely a strong and a weak one, and two different storylines. In one of the story conditions the player is tasked to approach the witch in the forest to capture her and in the other they are told to avoid the witch by running away. By providing different narratives, we aimed to elicit an implicit shift in the participants’ experience of the gamified task. However, because gamification can also have unintended consequences, such as exaggerating expectations by simply framing the task as a game [15], we refrained from in-game changes based on the narrative.

### 1.3 Hypotheses

Our primary hypothesis was that performance and subjective experiences change depending on the specific game-condition. Specifically, with regards to performance: we expected a better performance for people playing stronger avatars as indicated by a faster Stop-Signal Reaction time (SSRT), which is indicative of better performance in the task (akin to the Proteus-Effect; [31]. Additionally, due to the relation of avoidance motivation and inhibitory control, we expected that in line with the devaluation by inhibition hypothesis, participants will be faster to inhibit their response when an avoidance motivation is induced, when compared to an induced approach motivation as indicated by a lower SSRT [32].^2^

Further, based on earlier studies in which immersion has shown to enhance action control, we hypothesise that a deeper level of immersion will predict a faster SSRT [33,34]. Next to that, previous literature has shown that a greater avatar identification can enhance performance [[11], [13]]. Thus, we hypothesise that greater avatar identification leads to lower SSRT. Additionally, we hypothesised that a higher intrinsic motivation also predicts lower SSRT scores, based on a study that found a negative relationship between intrinsic motivation and mental fatigue (Herlambang et al., 2021).

## 2 Methods

### 2.1 Participants

To assess the sample size necessary to achieve a moderate effect size (f = .33) for a main effect or 2-way interaction, a power analysis was conducted using GPower [35]. Given a power of 1–β = .95, an α value of .05 and a moderate correlation between repeated measures (r = .5), a total sample size of 21 participants was calculated. Previous studies utilizing similar versions of the SSG employed samples ranging from 20 to 30 participants per group in a between subjects design or in repeated measures designs as an overall sample [1,7,36]. Although the studies did not find the originally hypothesised effect, the data indicates (including for example Bayes Factors) strong evidence for the null hypothesis and a sufficient sample to test it. Furthermore, other studies that reported a significant effect on performance in the SSG included similar samples [[8], [9], [11]]. A total of 45 undergraduate students have been recruited (26 female, 19 male) with a mean age of 21.48 (*SD =*2.81). They completed both session of the SSG and all rounds of questionnaires. Participants received course credit for their participation. They were made aware that credits would be given out regardless of their performance and that they would receive credits if they chose to abord the experiment if they felt too uncomfortable to continue. Most participants indicated being of Dutch nationality (*n* = 18), followed by a smaller group reporting being German (*n* = 14) or having another nationality (*n* = 13). The participants who reported having a different nationality were asked to specify this. To take part in the study, the only inclusion criteria was being 18 years old or above. Additionally, participants gave their informed consent at the start of the study and were made aware of their right to withdraw from the study at any time. The study was approved by the University of Twente BMS ethics committee (Approval Number: 230074).

### 2.2 Design

The study made use of a repeated-measures design to determine the impact of character conditions (“strong”, “weak”) and story condition (“approach”, “avoid”) on performance within participants. Therefore, we employed a 2 (character: weak/strong) x 2 (story: approach/avoid) mixed design. Every participant played two conditions per session, resulting in every participant having played all four possible conditions. Within one session the approach/avoidance condition remained the same, but the character changed for each participant. The independent variables of interest were the SSRT, as indicator for the time it took to cancel the already initiated movement and the Stop-Signal Delay (SSD), which is the delay between the go cue and the stop-signal that is based on participants error rates. Furthermore, the overall reaction time (RT) for Stop-Signal and non-Stop-Signal trials was assessed. Additionally, we looked at the overall go trial accuracy rates.

### 2.3 Materials

#### 2.3.1 Game design

**Fig. 1:**
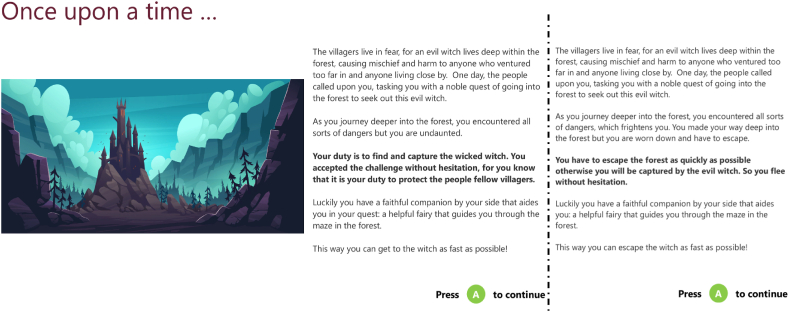
Instructions for the two different Story Versions. To the left the story version wherein the player is supposed to imagine approaching the witch in order to capture her. The story on the right has the player running away from the witch. Importantly the witch was not seen in the gameplay and the gameplay did not change depending on the story.

The SSG that participants completed, was a gamified version of the SST, that provided participants with a background story to make it more immersive (See Fig. 1 for details). The game was created according to recommendations for use of the SST by Verbruggen and colleagues, and has been previously validated [7,[37], [38], [39]]. The background story consisted of participants being told that they were stuck in an enchanted forest, and a fairy would help them to find the way by pointing left or right at every intersection. Once the fairy appeared on the screen, the participants had 1.5 s to react to the cue. Additionally, they were told that sometimes the fairy would be tricked by an evil witch to give the wrong directions. This could only be noticed by an audio cue in combination with a haptic signal (vibration) of the controller, appearing shortly after the fairy. In this case, they were told to ignore the advice of the fairy and not press anything.

**Fig. 2:**
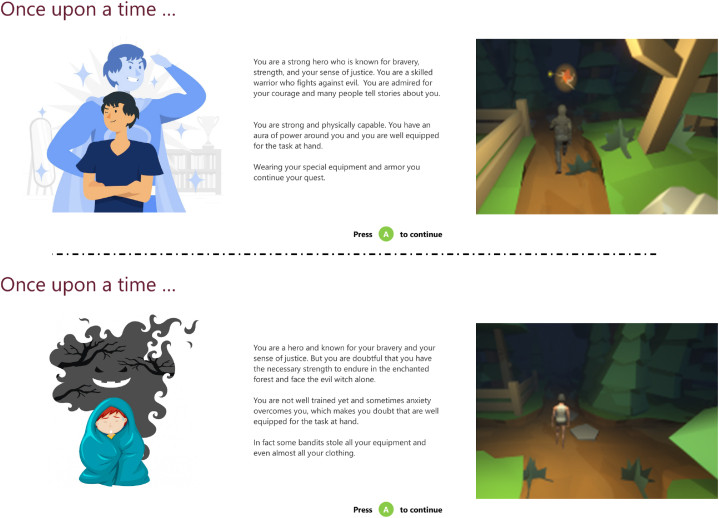
Top: Strong avatar backstory and representation in game. Bottom: Weak avatar and its depiction in the game.

Additionally, participants either played as a strong or weak character (see Fig. 2 for details). While the strong character was wearing a suit of armour in the SSG, the weak character was only depicted in a shirt and shorts. They further had different backstories associated with them.

#### 2.3.2 Stop signal task structure

A variety of performance measures were taken during their runs including the average RT of correct go-trials, omission and commission errors, the probability of participants to respond to a Stop-Signal trial and the average SSD as well as their average SSRT. The Stop-Signal delay was adjusted using a staircase method to adapt to the participants reaction speed. When the first Stop-signal occurred, participants had a delay of .25 s between the occurrence of the fairy and the sound. At subsequent encounters the delay increased by .05 s for each errorless Stop-Signal trial and decreased by .05 s for each trial without an error, so that the task became more difficult based on the skill-level of the participants. Furthermore, the intertrial interval was set to a random value between 500 msec and 1500 msec. This was done in order to reduce the likelihood of participants falling into a response rhythm, wherein each trial starts at the same time after the last. Every session consisted of 200 trials in total, of which 75 % were ‘go’ and 25 % were ‘stop’ trials. There were two 15 s breaks during each session. As mentioned previously, the time in between the go- and Stop-signal adjusts to the performance of the participants. This was done with the staircase procedure in order to obtain a probability of inhibiting the button press of 50 %.

#### 2.3.3 Control questionnaires

**Barratt Impulsiveness Scale (BIS-15).** The BIS-15 was used to assess the participant's personality trait of impulsiveness. The scale examines three facets of impulsivity – namely non-planning, motor, and attentional impulsivity – through 15 statements to determine a measure for general impulsivity [40]. Non-planning impulsivity mainly measures an individual's ability for cautious thinking and planning through items like ”I plan tasks carefully” [40]. Moreover, motor impulsiveness assesses an individual's inclination towards spur of the moment behaviour and lifestyle consistency, with items such as “I buy things on impulse” [40]. Lastly, attentional impulsiveness evaluates “task-focus, intrusive thoughts, and racing thoughts” via items similar to “I am restless at the theatre or lectures” [40]. Each statement is rated by the participant using a bipolar 4-point Likert scale ranging from ‘Never’ to ‘Always’.

**Behavioural Inhibition System/Behavioural Activation System (BIS/BAS) Scale.** The BIS/BAS scale by Carver and White [28], is the most widely used questionnaire for assessing the BIS and BAS [41]. This scale was used to measure trait-based approach/avoidance motivation of the participants, before it was attempted to experimentally manipulate this through the story behind the game task. The BIS/BAS scale includes 20 items and consists of four subscales. Three of the subscales correspond to different aspects of the BAS, namely BAS Drive, which is the motivation to follow one's goals, consisting of four items, BAS Reward Responsiveness, measuring how sensitive one is to pleasant reinforcers from the environment which consists of four items as well. Finally, BAS Fun Seeking measures how motivated people are to spontaneously seek novel rewards and consists of five items. The remaining seven items correspond to measuring the BIS, or avoidance motivation. Each item was scored on a 4-point Likert scale, with the options “Strongly agree”, “Agree”, “Disagree” and “Strongly disagree” [28].

#### 2.3.4 Gamification questionnaires

**Immersion Questionnaire.** The immersion questionnaire used in this study was developed by Jennet et al. [42] to quantify the experienced immersion in gamified tasks. The questionnaire consists of 32 questions which are answered on a 7-point Likert scale ranging from “Strongly disagree” to “Strongly agree”. The scores of every second item had to be reversed, since they were a negative version of the preceding item and thus indicate that the participant was not immersed in the game. Additionally, the immersion questionnaire includes one question that is answered on a slider from 1 to 10 to indicate how immersed the participant felt overall, ranging from “Not at all immersed” to “Very immersed”. The immersion questionnaire was used after each of the four conditions was played. Higher scores indicate that the participant was more immersed in the SSG.

**Player Identification Scale (PIS).** To measure a participant's identification with an avatar, the PIS was used [43]. The used scale consists of three subscales, namely the similarity identification, embodied identification, and wishful identification subscales. Similarity identification aims to measure the degree of similarity observed by the player between themselves and their avatar using a total of six statements presented, an example being “My character resembles me”. Embodied identification measures the extent of the player feeling like they are the avatar while playing the game through six statements, such as “In the game, it is as if I become one with my character”. Wishful identification aims to assess the extent of the desire of a player to resemble their avatar more using five statements such as “I would like to be more like my character”. The participants could rate their agreement with each statement using a 5-point Likert scale ranging from “Strongly agree” to “Strongly disagree” [43].

**Intrinsic Motivation Inventory (IMI).** The IMI was used to determine participant's intrinsic motivation of completing the task. The shortened version of the IMI was used to measure the following sub-facets: interest/enjoyment, perceived competence, perceived choice, and pressure/tension [44]. Interest/enjoyment assesses how enjoyable the task experience was for participants and is presented with items like “Doing the task was fun” or “I thought the task was very interesting”. Perceived competence refers to how well participants felt they did on the task. One of the items used was “After working at this task for a while, I felt pretty competent”. The perceived choice subscale used items like “I felt that it was my choice to do the task” to determine if participants felt like they had influence on the task and their choices mattered. Lastly, pressure/tension measured how they perceived pressure or anxiety while completing the task. Participants were asked to indicate their agreement with the statements presented using a 7-point Likert scale ranging from “Not true at all” to “Very true”.

### 2.4 Procedure

Participants were sampled through SONA-systems. On SONA-systems, the participants received additional information about this study and could volunteer to participate by signing up for two different time slots on different days. Upon entering the laboratory, participants were seated in front of a PC with Windows 11 equipped with Sennnheiser HD 201 or JBL Quantum 400 headphones (overall attenuation for Sennheiser: −7.23 dB and for JBL: −14.94 dB). In previous pilots it was determined that a fixed stop-signal volume can be uncomfortable for some participants depending on the thresholds, thus the volume was adjusted for each participant and the experimenters made sure that it was audible over the headphones. The average loudness of the stop-signal was approximately 60 dB. The tactile part of the stop-signal was presented via an Xbox Elite gaming controller. The tactile signal lasted for .5 s and had a frequency of 250 Hz with a peak-to-peak amplitude of about 128 mm. The standard viewing distance from the computer was approximately 60 cm. Next, participants received information about the session procedure and were given an informed consent form to sign. Participants additionally received information that they had to react as fast as possible to the cues and that the game adapts its difficulty to their reaction time, so that the task does not become too easy nor too difficult to respond appropriately to. Following the instruction, participants were firstly asked to fill in a short questionnaire about demographic variables, such as age, gender, and nationality on the online survey platform Qualtrics. After that, the participants had to fill in the BIS-15 [40] and the BIS/BAS scale [28], which served as control questionnaires for individual differences.

Afterwards they had to play two of the four possible conditions of the SSG. In the first session, participants either played the approach or the avoidance condition that was introduced through a short background story in a tutorial, in which participants could practise the controls of the game. In the approach condition participants received a story in which the goal was to find and capture a wicked witch to protect the villagers. In the avoidance condition participants were told to flee and escape from the wicked witch. For each participant, the condition was randomised between the first and second session in order to reduce practice effects. In addition to the story, the tutorial also introduced the character to be used by the participant for that specific trial. Within each session the approach or avoidance condition stayed the same and were played twice. Once with a strong character, that was framed as a strong hero that is well physically trained and has strong armour, and once with a weak character, that was framed as a hero with only little training whose armour was stolen by bandits. The optic appearance of the character changed based on the different descriptions. In the tutorial, participants completed 20 test trials that were not recorded. After completing the tutorial, participants played four blocks of the recorded trials consisting of 50 trials each (200 trials in total). Between each 50 trials block, participants had 15 s to rest.

In each of the conditions the task itself stayed the same. In the SSG, the character moved by itself through a forest. At each crossing, participants encountered a fairy that indicated the direction in which they had to move the character, by either pressing the left or the right bumpers on an Xbox controller. When a haptic signal of the controller occurred in combination with an audio cue in their headphones participants were instructed not to press any button regardless of the direction in which the fairy pointed. Importantly, during the explanation of the procedure of the study, the participants were explicitly made aware that the stop signal delay was dependent on their performance and that they should respond as quickly as possible to the visual cue on the screen and not to wait for the stop-signal. They were also explicitly made aware that it would not always be possible to respond correctly to the stop-signal because of this adjustment of the stop-signal delay. Furthermore, participants were also observed during the tutorial in order to spot any person that tries to “game the system”. They were also repeatedly reminded about the task rules (i.e. react as fast and as accurate as possible and stop the already initiated action when the stop signal appears) throughout the sessions.

After each block, participants were asked to complete the immersion questionnaire [42], the PIS Van [43] and the IMI [44] to quantify their experience of the game. In every session the three questionnaires were filled in twice, once for the strong character trial and once for the weak character trial. When both trials had been completed and the questionnaires had been filled in twice, participants were free to leave and come back for the next session a few days later, where the procedure was repeated for the remaining condition. The second session differed from the first insofar as they did not have to fill in BIS-15/BAS scale or provide their demographics again. Each session lasted for about 1 h.

### 2.5 Data analysis

To begin, the data was filtered to remove faulty or invalid participant data; see *Data reduction* section for an in-depth explanation of this process. Subsequently the validity of the obtained remaining data was investigated. The general relationship that was investigated is whether the different game like features, namely the story versions and character conditions, influence the approach or avoidance motivation of participants and consequently their ability to stop the already initiated button press for the stop-trials. Further relationships were conducted to control for the effect of impulsiveness and personality-based BIS/BAS orientation on an individual's ability to inhibit already initiated behaviours. Lastly, the effect of avatar identification, immersion and flow/enjoyment on performance were investigated.

### 2.6 Data reduction

For the data to be able to measure what is intended to measure, some specific exclusion criteria related to the task performance of the participants were revised. Firstly, participants who did not complete all four correct avatar-storyline variations were excluded from the dataset. Secondly, participants were excluded from the data if their p(response|signal) was below .25 or above .75 for any of the four condition combinations, as this might be an indication that participants waited for the stop-signal to occur, which biases the outcome measures [37]. Thirdly, it was determined that the accuracy rate for participants pressing the correct button during a go trial (e.g., left or right) to turn towards the direction the fairy indicated should be at least 80 % or higher to be retained in the dataset, as high error rates can further bias outcome measures [37]. Next, the horse-race test variable in the dataset was assessed for every participant. According to the horse-race assumption, the RT for unsuccessful stop trials needs to be lower than the mean go-RT to reliably estimate the SSRT [45]. Therefore, the participants for which the horse-race test variable gave a negative value for any of the four condition combinations were removed from the dataset altogether. Then, the questionnaire data was assessed to see whether all the responses were recorded in the dataset. Lastly, the variance for each participant was evaluated to identify flatliners. Based on these exclusion criteria, a total of eleven participants were excluded from the data, leading to a final sample size of 32 subjects (14 male and 18 female, *M(Age*) = 21.24, *SD* = 1.82). Participants indicated being mainly Dutch (*n* = 12), followed by the category German (*n* = 9) and other (*n* = 11).

## 3 Results

### 3.1 Preliminary data analysis

After checking the race-test assumption for each person in each session it is further recommended to check for a significant difference between correct go-trial RT and incorrect stop-signal trial RT for all experimental conditions [37,45]. A 2 (Trial type: signal/no signal) x 2 (Character: strong/weak) x 2 (Story: approach/avoidance) mixed analysis of variance resulted only in a significant difference between signal and no signal trials (F_Trial_type_(1, 124) = 310.98, *p* < .001, η_p_^2^ = .018), indicating that the data gathered by the SSG is valid. For the story and character conditions, no significant main effects or interaction effects were observed (for all *p* > .05).

With regards to the questionnaires, Cronbachs alpha was calculated as a measure of internal consistency. Participants responses to the inhibition (M = 2.89, SD = .46) and activation scales (M = 2.94, SD = .38) of the BIS/BAS questionnaire were assessed on a 4-point-Likert scale. The inhibition scale shows an acceptable reliability (α = .78), while the activation scales overall show a high reliability (α = .83). Questions on the BIS-15 (M = 2.33, SD = .37) were also answered on a 4-point Likert scale and overall showed good reliability (α = .85). Reliability coefficients of the Immersion questionnaire (α = .92), PIS (α = .93), and IMI (α = .94) overall showed very high reliability. Because the BIS/BAS and BIS-15 were only filled in once, these calculations are based on 32 observations while the calculations for the Immersion questionnaire, the IMI and the PIS were based on 128 observations as they were filled in after each of the four conditions. The descriptive statistics for all scales, including subscales, and performance measures can be found in Table 1.

**Table 1:** Descriptive statistics of the Immersion Questionnaire, Player Identification Scale (PIS), Intrinsic Motivation Inventory (IMI) and Performance.

Variable	Range	Session 1	Session 2	Strong-Approach	Strong-Avoidance	Weak-Approach	Weak-Avoidance	Total
		M(SD)	M(SD)	M(SD)	M(SD)	M(SD)	M(SD)	M(SD)
Immersion	1–7	3.81(.77)	3.36(.92)	3.61(.96)	3.56(.85)	3.51(.89)	3.66(.82)	3.59(.87)
PIS	1–5	2.02(.73)	1.91(.86)	2.20(.91)	2.08(.83)	1.84(.72)	1.72(.65)	1.96(.80)
IMI	1–7	4.09(.61)	3.70(.73)	4.05(.62)	3.80(.74)	3.82(.70)	3.92(.73)	3.90(.70)
SSRT (ms)	134.88–568.82	276.71(84.26)	291.13(82.46)	284.60(93.19)	286.52(78.32)	289.23(83.28)	275.32(81.20)	283.92(83.35)
SSD (ms)	42.71–941.67	325.26(197.53)	332.32(186.48)	353.35(219.32)	309.73(196.65)	344.14(175.51)	307.94(175.50)	328.79(191.36)
Correct go RT (ms)	357.71–1320.00	627.73(213.02)	645.52(211.60)	665.55(236.48)	615.69(216.65)	660.00(203.78)	603.26(189.97)	636.13(211.64)
Accuracy rate (%)	87.50–100	98.63(2.13)	98.63(1.75)	98.56(2.42)	98.54(1.98)	98.48(2.01)	98.95(1.19)	98.63(1.94)

### 3.2 Experimental condition effects on task performance

In order to assess the impact of personality on performance we correlated the BIS/BAS questionnaire as well as the BIS-15 with the performance measures in the task. Results revealed a significant correlation of the Fun Seeking subscale of the BIS with SSRT (r(30) = .45, *p* = .011) indicating a higher score on the Fun Seeking scale would lead to worse performance. Further, all subscales of the BIS-15 as well as their total score correlated positively with SSRT (r's = .36 - .51, p < .05), indicating worse performance for higher impulsivity individuals. Table 2 contains further correlations of personality measures and task performance. Overall, these results were expected. As a consequence in the following analysis we needed to control for these variables. Contrary to the primary hypothesis, a linear mixed model with avatar and story condition as independent variables and participant ID, overall impulsiveness and fun seeking as random factors resulted in no difference between the conditions regarding SSRT [F_Avatar_(1, 93) = .08, *p* = .782; F_Story_(1, 93) = .26, *p* = .613; F_Avatar:Story_(1, 93) = .45, *p* = .505] and accuracy rate [F_Avatar_(1, 93) = .368, *p* = .546; F_Story_(1, 93) = .70, *p* = .407; F_Avatar:Story_(1, 93) = .83, *p* = .365]. However, for correct go-trial reaction time [F_Avatar_(1, 93) = .17, *p* = .685; F_Story_(1, 93) = 5.84, *p* = .018, η_p_^2^ = .059; F_Avatar:Story_(1, 93) = .02, *p* = .876] a significant effect of story emerges and for SSD [F_Avatar_(1, 93) = .09, *p* = .770; F_Story_(1, 93) = 4.55, *p* = .036, η_p_^2^ = .047; F_Avatar:Story_(1, 93) = .04, *p* = .843] this main effect of story is significant as well.

**Table 2:** *Correlations of Stop-Signal Reaction Time (SSRT), Stop-Signal Delay (SSD), correct go trial mean reaction time (RT) accuracy, Behavioural Inhibition and Activation (BIS/BAS) and Impulsivity*. Calculations are based on 32 observations. To correlate SSRT, SSD, go trial RT and Accuracy with Impulsivity and Behavioural Activation and Inhibition the mean values per participant of all 4 sessions were calculated. ∗p < .05. ∗∗p < .01. ∗∗∗p < .001.

Variable	M	SD	1	2	3	4	5	6	7	8	9	10	11	12
1. SSRT(ms)	283.92	61.12	–											
2. SSD (ms)	328.79	169.34	−.15	–										
3. Accuracy (%)	98.63	1.43	−.**57∗∗∗**	.22	–									
4. Go trial RT (ms)	636.13	182.87	.20	**.94∗∗∗**	.00	–								
5. BIS	2.89	.46	−.12	.01	−.17	−.01	–							
6. BAS (Total)	2.94	.38	.26	.03	−.27	.14	.26	–						
7. BAS (Drive)	2.56	.50	.10	.10	−.14	.15	.05	**.79∗∗∗**	–					
8. BAS (Reward)	3.21	.45	.10	−.02	−.19	.03	**.46∗∗**	**.81∗∗∗**	**.42∗**	–				
9. BAS (Funseeking)	3.00	.47	**.45∗**	.01	−.32	.19	.07	**.81∗∗∗**	**.51∗∗**	**.48∗∗**	–			
10. Impulsivity (Total)	2.33	.37	**.51∗∗**	−.15	**−.48∗∗**	.04	−.33	.33	.17	.16	**.48∗∗**	–		
11. Impulsivity (Motor)	2.34	.39	**.42∗**	−.16	**−.41∗**	.01	−.20	**.39∗**	.30	.21	**.45∗**	**.84∗∗∗**	–	
12. Impulsivity (Non planning)	2.14	.54	**.45∗∗**	.06	−.31	.22	**−.48∗∗**	.10	.01	−.12	**.39∗**	**.83∗∗∗**	**.54∗∗**	–
13. Impulsivity (Attention)	2.53	.42	**.36∗**	−.32	**−.48∗∗**	−18	−.05	**.37∗**	.16	**.37∗**	**.36∗**	**.78∗∗∗**	**.59∗∗∗**	**.38∗**

### 3.3 Session effects on performance

Linear mixed models with session as predictor and participants, impulsivity and fun seeking as random factors to control for within-participant variance resulted in no significant effect of session on SSRT [F(1, 96) = 1.55, p = .216], SSD [F(1, 96) = .14, p = .709], go trial RT [F(1, 96) = .57, p = .454] or accuracy [F(1, 96) = .00, p = 1], so there was no indication of practice effects. However, the linear mixed model with immersion as dependent variable, resulted in a significant decline in immersion from session 1 to session 2 [F(1, 96) = 28.36, p < .001, η_p_^2^ = .228]. Similarly, intrinsic motivation significantly declined from session 1 to session 2 [F(1, 96) = 14.36, p < .001, η_p_^2^ = .130], suggesting that participants became significantly less motivated and immersed over time. For avatar identification no significant effect of session was found [F(1, 96) = .88, p = .350].

### 3.4 Motivational changes due to gamification and their effects on performance

**Fig. 3:**
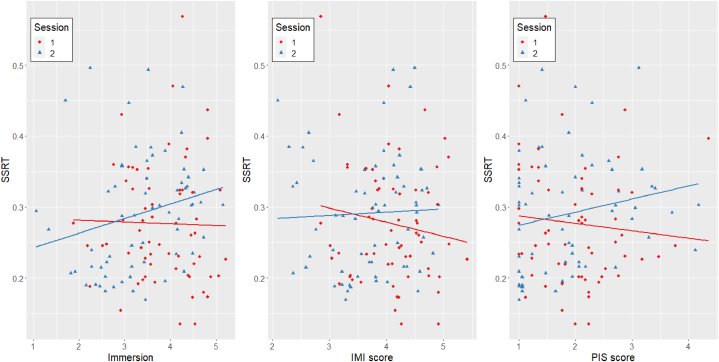
Interaction effects of Session with Immersion, Intrinsic Motivation and AvatarH1 Identification on Stop-Signal Reaction Time (SSRT). For a table of bivariate correlation between SSRT and the individual questionnaire scores, split by session refer to the supplemental materials.

To assess the impact of the changes in the game on motivation and performance we correlated the IMI, immersion and PIS questionnaires with the performance measures (see Table 3). Subsequently, we carried out linear mixed model analysis to quantify the potential effect of motivation on performance. Against our hypothesis, a linear mixed model with immersion as predictor and participant ID, impulsiveness and fun seeking as random factor variables resulted in a significant positive effect on SSRT [F(1, 111.68) = 4.19, p = .043, η_p_^2^ = .036], meaning the more immersed participants were, the slower they reacted to the stop signal. The same model with overall avatar identification mean score resulted in no significant effect on SSRT, suggesting that avatar identification did not predict performance in the SSG [F(1, 125.81) = .87, p = .353]. Next to that, a linear model was used to explore whether the gender of a participant influenced the avatar identification, as the played avatar had a more male appearance. This, however, did not significantly affect the avatar identification score (t(30), = −1.479, p = .15). Similarly, overall intrinsic motivation scores did not predict SSRT, suggesting that motivation did not affect performance in the SSG [F(1, 121.49) = .05, p = .831]. However, the effect of immersion but not intrinsic motivation is mediated by session (Fig. 3), so that its’ effect on SSRT can be explained by a significant increase in predictive power from session 1 to session 2 [F_Session_(1, 98.39) = 1.93, p = .167; F_Immersion_(1, 110.10) = 7.24, p = .008, η_p_^2^ = .062; F_Session:Immersion_(1, 97.46) = 3.99, p = .049, η_p_^2^ = .039].

**Table 3:** Correlation matrix of Stop Signal Reaction Time (SSRT), Stop Signal Delay (SSD), Accuracy, go trial reaction time (RT) and Immersion, Intrinsic Motivation Inventory (IMI) and Player Identification Scale (PIS) scores. Calculations are based on 128 observations (4 per participant). ∗p < .05. ∗∗p < .01. ∗∗∗p < .001.

Variable	Mean (SD)	1	2	3	4	5	6	7	8	9	10	11	12	13
1. SSRT	283.93 (83.35)	–												
2. SSD	328.79 (191.36)	−.10	–											
3. Accuracy	98.63 (1.39)	**−.41∗∗∗**	.17											
4.Go RT	636.12 (211.64)	**.29∗∗∗**	**.92∗∗∗**	−.02										
5. Immersion	3.59 (.87)	.09	−.05	−.00	−.01	–								
*Intrinsic Motivation Inventory*														
6. Total	3.90 (.70)	−.07	.01	**.19∗**	−.02	**.54∗∗∗**								
7. Joy	3.99 (1.39)	−.04	−.04	.10	−.06	**.48∗∗∗**	**.85∗∗∗**	–						
8.Competence	3.59 (1.20)	−.04	.01	.17	−.01	.15	**.49∗∗∗**	**.20∗**	–					
9. Choice	5.37 (.90)	−.01	−.11	.04	−.11	**.21∗**	**.44∗∗∗**	**.33∗∗∗**	.17	–				
10. Tension	2.64 (1.32)	−.05	.14	.12	.12	**.36∗∗∗**	**.48∗∗∗**	**.35∗∗∗**	**−.19∗**	**−.25∗∗**	–			
*Player Identification Scale*														
11. Total	1.96 (.80)	.05	−.09	.08	−.06	**.29∗∗**	**.41∗∗∗**	**.28∗∗**	**.36∗∗∗**	.14	.15	–		
12. Similarity	1.83 (.88)	.02	−.00	.06	.00	**.20∗**	**.27∗∗**	**.20∗**	**.26∗∗**	.08	.08	**.88∗∗∗**	–	
13. Empathy	2.27 (1.10)	.05	−.16	.07	−.14	**.34∗∗∗**	**.45∗∗∗**	**.30∗∗∗**	**.39∗∗∗**	.04	**.25∗∗**	**.83∗∗∗**	**.59∗∗∗**	–
14. Wishful Identification	1.74 (.94)	.06	−.02	.06	.00	.13	**.24∗∗**	.16	**.20∗**	**.26∗∗**	−.01	**.73∗∗∗**	**.57∗∗∗**	.32

Interaction of gamification effects and experimental conditions.

A linear mixed model with story version and avatar condition as independent variables and participants, impulsivity and fun seeking as random factor variables had no effect on immersion [F_Story_(1, 96) = .26, p = .612; F_Avatar_(1, 96) = .00, p = .988; F_Story:Avatar_(1, 96) = 1.07, p = .304] and overall intrinsic motivation [F_Story_(1, 96) = .59, p = .445; F_Avatar_(1, 96) = .25, p = .615; F_Story:Avatar_(1, 96) = 2.60; p = .110], suggesting that the experimental manipulations did not have any effect on immersion and the intrinsic motivation of participants. The mean avatar identification, however, was significantly lower for the weak avatar than for the strong avatar [F_Avatar_(1, 96) = 11.31, p = .001, η_p_^2^ = .105] but there was no difference between the story conditions or the interaction effect of avatar and story[F_Story_(1, 96) = 1.28, p = .260; F_Story:Avatar_(1, 96) = .00; p = .986].

## 4 Discussion

### 4.1 Summary of results

This study aimed to investigate the effects of gamification on response inhibition in the SST and to quantify how participants experienced the gamification. The results show that while controlling for fun seeking and impulsiveness, higher immersion predicts worse response inhibition, especially in the second session. This effect could be explained by a significant decline of immersion from the first to the second session. The effects of intrinsic motivation and avatar identification did not significantly predict SSRT, yet both declined going from the first to the second session suggesting that participants overall had a worse gaming experience in the second session. However, the consistent negative relationship between immersion and SSRT in session 2 highlights that contrary to our original hypothesis, increased immersion can lead to poorer response inhibition. This finding suggests that while gamification elements might initially enhance engagement, they may also draw attention away from critical task components, ultimately impairing performance. Next to that, the approach and avoidance narratives did not affect any of the outcome variables or the gaming experience of participants, but the different playable avatars altered participants’ avatar identification as they could identify more with the strong avatars. The narratives of the game did not have the hypothesised effect on the experience nor the performance variables, while the avatar strength only influenced the extent to which people could identify with the avatar but no other variables. Hence, the distinction between the different game elements was too implicit to induce any relevant changes within participants. Furthermore, the data reveals a negative correlation between immersion and inhibitory control.

### 4.2 Theoretical implications

In this case, a gamified version of the SST allows researchers to enhance the ecological validity of the inhibition measurement by presenting the task in a visually complex environment, while also keeping participants motivated to perform well. Further, ideally due to the addition of certain game-like elements motivation may be enhanced (or reduced), which may lead to an improvement (or reduction) in performance.

Session 1 revealed no significant correlation between SSRT and immersion. However, the pattern of effects for session 2 is different. Although overall immersion decreased from session 1 to 2, the correlation reversed directions. Thus, higher immersion in session 2 led to reduced inhibitory control. Thus, although generally immersion was lower in session 2, the people that were more immersed did perform worse. This may be due to a shift from an active player that wants to “beat the game” to a more detached, less immersed observer that more passively follows the story. Similarly, for avatar identification, as measured with the PIS, the negative correlation with SSRT in session 1 turned positive in session 2. Thus, stronger identification led to better inhibitory control in session 1 but worse inhibitory control in session 2. Overall, this challenges the assumption that gamification universally benefits cognitive tasks. The general decline of engagement from session 1 to session 2 could be due to factors like the task becoming less novel or participants experiencing fatigue, especially considering the prevalence of engaging video games outside the lab setting. With regards to motivation, the data revealed a negative association between IMI scores and SSRT in session 1, meaning higher IMI scores lead to better performance. However, in session 2, this relation was nullified. Thus, the pattern of effects for avatar identification and immersion are comparable across sessions, while motivation seems to only affect performance in the first session. Comparatively, the present game-like task is still arguably more monotone than a standard infinite runner (e.g., Metroland or Subway Surfer). Thus, an arguably theoretically intriguing, explanation for the session 1 findings is that they represent a genuine effect but masked by session 2. Hence, in the future incorporating more dynamic and continuous measures of user engagement alongside SST by for example making use of physical or digital sliders [46]. Furthermore, individual differences in personality traits related to novelty-seeking or boredom susceptibility might influence this relationship, but they were not assessed in the present study. By systematically addressing these factors, we can gain a deeper understanding of how user experience modulates inhibitory control.

This ties into the proposition that modern technology can be used to enhance mundane and experimental realism while keeping experimental control high and potentially even increase the effect of experimental manipulation [47,48]. Thus, it is important to choose a task design that not only reliably taps into the targeted processes (i.e. the response inhibition process) but also leads to increased participant engagement [49,50]. Based on the present results, we suggest future researchers that wish to iterate on the present task design, to implement several changes to increase the likelihood of manipulating motivation and performance. Here, only the BAS Fun Seeking subscale significantly correlated with participants' performance, while BIS and other BAS subscales did not. This lack of correlation suggests that certain aspects of gamification may fail to activate approach/avoidance motivation effectively. This may have been because there were no tangible consequences for failure or success in the task. For example, if performance was poor the player was not caught or attacked by the witch. In future research, additional mechanics should be implemented to further strengthen this sense of urgency and danger. This would be in line with a previous study reporting that response inhibition was impaired when a potential threat occurred during stop trials [51]. However, in their study the distractor used was present during the trial itself, while in our study, the threat was only presented in the background story. For the threat to have an impairing effect on response inhibition, an emotional signal needs to actively compete for available cognitive resources [51].

An alternative explanation for the findings of the current study, however, could be that the consistent negative correlations of immersion and avatar identification to SSRT in session 2 were genuine and masked by the effects of session 1. In the first session participants might have attempted to “beat” the game, meaning they were more focused on inhibiting their response. In session 2, however, this changed so that more of the participants' attention was drawn to other aspects of the game (i.e. graphics, story etc.) so that motivational aspects like immersion and avatar identification might have drawn attention away from relevant task elements (response inhibition). This explanation is in line with the finding that a failure to stop prepared motor responses is linked to how attention is allocated in advance of the go and stop stimuli [52]. When participants' attention is more focused on the immersive elements of the gamified task rather than the task's inhibitory demands, their performance may suffer as a result. In the past it has also been shown that the participants' emotional state [53] and the infrequency of the stop signals [54] can lead to impaired performance, which further may complicate experimental control in gamified tasks. Therefore, it is important to design engaging game-like features for a task and ensure that attention is not directed towards task-irrelevant aspects.

### 4.3 Practical implications

The decline in motivation over time could be explained by the fact that gamification elements such as a narrative to induce approach and avoidance motivation, graphic elements that fit the theme of the story and different playable avatars were given but for the participants it did not feel like playing a game. This may be caused by a lack of a concrete goal within the task, so that the narrative alone was not strong enough to induce a goal motivation in participants [55]. A certain amount of complexity or a problem for the player to solve may be needed to engaged participants effectively [56]. The SSG that was used in this study might have lacked this complexity and did not offer opportunities for autonomy or problem solving so that this might have caused participants to become less immersed and motivated over time. Inadvertently this may have also subverted the expectations of the participants, due to the labelling as a Stop-Signal *Game* [25,26].

Any future change in the game design also be more effective when the identification with the in-game avatar is stronger by for example harnessing the self-prioritization effect or allowing for avatar customization [11,20]. This reinforced connection between player and avatar, may yield a Proteus-Effect like result [31]. The model proposes that participants use identity cues, such as an avatar's appearance, to infer how they expect an avatar containing the observed characteristics to behave, modifying their behavioural responses and attitudes accordingly. However, if identification with the avatar is not strong enough, the Proteus Effect can't come to fruition. Thus, for example, in our study, the strong avatar was described and presented in the game as being physically well trained and wearing strong armour, while the weak avatar was robbed from his armour by bandits and physically presented as having received only little training. In the strong avatar condition would then either perform better to match the depiction of the character in the game, or they may feel too confident and make more mistakes than otherwise.

An additional avenue to explore in the future involves increasing the interactivity of the story, as the story in the current study was not distinct enough. For example, during task breaks the participants may need to make story choices that may or may not actually have a consequence for future gameplay. The act of choosing has been shown to increase transportability into the narrative and identification with the character [13,14]. Thus, in turn such a manipulation may inadvertently increase any manipulation based on the avatar.

The present results may also inform our understanding of the underlying stopping process considering that they suggest that the stop- and the go-process as well as influences on those are not as separate as typically suggested by the horse-race model. This further may inform potential interventions in the future. Case in point, one of the more consistent results in SST research is that Attention-Deficit-Hyperactivity-Disorder (ADHD) has been associated with reactive response inhibition deficits. With successful medication these individuals can perform adequately on response inhibition tasks such as the SST [57]. However, specifically, these seem to be traced back to deficits in early attentional processes, rather than inefficiency in the stop process [58]. Hence, in ADHD, response inhibition is poor, especially because of early attentional processes, for which game-elements might be helpful or detrimental. Nonetheless, it is generally unclear how game elements and motivational factors, such as immersion affect attentional processes in individuals with impulse control problems. It has for instance been shown that deficits in inhibitory control are preserved for overweight individuals in a gamified SST, which might have been due to an attentional shift to irrelevant game elements [59]. Yet, in a food related gamified SST, response inhibition positively correlated with food-craving and hunger for a high-calorie version compared to a low-calorie or non-food version of the task [1]. Thus, momentary attentional shifts linked to in-game stimuli can impact performance. Knyazev and colleagues [52] further conclude that response inhibition fails if attention is more directed towards task-irrelevant stimuli while preparing for stopping a response. Therefore, gamification elements introduced to a task or intervention need to tap into the relevant cognitive processes, such as response inhibition in the SST in order to be effective.

### 4.4 Limitations

One important limitation of this study is that the results may not be generalisable to relevant populations, since the sample exclusively consisted of undergraduate students. This may be important because the measurement of action control tendencies such as response inhibition and approach-avoidance motivation are often applied in a clinical setting e.g. [60], [61]. Future research should further explore the influence of avatars and story-driven changes in action control by for example, providing initially powerful avatars that are later removed in order to provide an accentuated power-difference, or allowing for avatar customization. Additionally, implementing additional immersive elements, such as incorporating user choices influenced by perceived avatar strength, could be beneficial. Finally, replacing the scenario (e.g., the witch) with one more relevant to participants' experiences (e.g., spiders for arachnophobes) might enhance engagement and provide more meaningful insights into how people perceive and react to self-relevant stimuli. By incorporating these future directions, research on gamification's role in influencing action control tendencies across various populations can be significantly strengthened. Another limitation of the current study is the lack of a pure control condition. In the present study participants played through all possible conditions of the task but never a version without any game-like elements. Thus, although we utilized a fully repeated measures design, only conclusions about the differences in game-conditions can be drawn but not about a general performance alteration in relation to the base-task. This would need to be explored in future work.

With regards to the chosen scales, the immersion questionnaire by Jennett et al. [42] only allowed the overall assessment of immersion, while disregarding different subdimensions of immersion that might explain in more detail which elements of immersion were associated with a decline in performance. Assessing the effects of different sub-dimensions of immersion, such as temporal dissociation, focused immersion, emotional involvement and transportation, may reveal more nuanced information about how immersion influences performance [42].

Another limitation of the present study are the unintended performance adjustments by participants that may, or may not, be supportive of the initial hypothesis but in any case, mask an effect of task condition. Exceedingly long response times and SSDs were recorded for some participants even though they overall provide valid data. It may be that those individuals deliberately slowed down their reaction so they could more readily inhibit their reaction. Although, participants not adhering to task rules is not a new discovery (e.g., earliest examples include people squinting in order to blur their vision when doing the Stroop task), this danger may be exacerbated for game-like tasks. Although game-like tasks can enhance performance and even make it more consistent, they may also influence performance in an undesired way (e.g., [62], [63], [64], [65]). Thus, there is a fine balance to be struck between the desired and undesired effects of gamification; for example, a task may want to be motivating enough to increase performance consistency but not motivating enough to alter performance. Following this line of reasoning it may be that because of the avatar and story manipulation in the study this behavior was expressed more; unfortunately to the detriment of our results and specifically SSRT estimation. Thus, it may have been that the experimental manipulation was more effective then the results show at first glance and some participants were intentionally slowing down to avoid inhibitory errors on stop-signal trials, but the current data cannot provide conclusive evidence for this as the experimental setup is not sensitive enough for this.

## 5 Conclusion

The present study shows that light-touch gamification can have an impact on motivation and performance One possible explanation for a decline in motivation over time may be that participants were expecting a more grandiose game with more game-like elements (e.g., a scoring system, combat with the witch, avatar customization) and the novelty aspect of the simple game-elements quickly faded during the second session. Alternatively, the game like elements that were employed in the SST could have directed attention away from the tasks' goal to inhibit their response, explaining a negative correlation between motivational variables and task performance. Therefore, researchers must tread carefully when employing gamification elements, as the balance between enhancing motivation and undermining the task's authenticity can be delicate. Striking this balance requires a nuanced understanding of the cognitive and emotional dynamics involved, as well as a keen awareness of the potential consequences of gamification. In some cases, the superficial layer of gamification might inadvertently steer participants away from the task's underlying objectives, leading to a misalignment between researchers' intentions and participants' actual experiences.

## CRediT authorship contribution statement

**Linus Held:** Writing – review & editing, Writing – original draft, Visualization, Project administration, Methodology, Investigation, Formal analysis. **Johannes Pannermayr:** Writing – review & editing, Writing – original draft, Visualization, Project administration, Methodology, Investigation, Formal analysis. **Alina Kaufmann:** Writing – review & editing, Writing – original draft, Visualization, Methodology, Investigation. **Marouscha Scheffer:** Writing – original draft, Visualization, Formal analysis, Data curation. **Paola Flores:** Writing – original draft, Visualization, Formal analysis, Data curation. **Martin Dechant:** Writing – review & editing, Writing – original draft, Software, Methodology, Conceptualization. **Maximilian A. Friehs:** Writing – review & editing, Writing – original draft, Visualization, Validation, Supervision, Software, Methodology, Formal analysis, Data curation, Conceptualization.

## Data availability statement

Can be accessed free of charge at https://osf.io/d4yws/.

## Material and code availability statement

Can be accessed free of charge at https://osf.io/d4yws/.

## Declaration of competing interest

The authors declare that they have no known competing financial interests or personal relationships that could have appeared to influence the work reported in this paper.

## Footnotes

1 For other examples of light-touch gamification please refer to for example: in relation to stopping performance [1] or to probe social anxiety [2].

2 In a study on controlling behavior, the researchers investigated whether people would simply do less of everything (global suppression) or if controlling urges would make them less likely to pursue previously desired things (devalued by inhibition) but more likely to avoid unpleasant things. The study found the latter, suggesting people don't become generally inactive after controlling urges, but rather more selective in their actions. Put differently, participants were less likely to engage with a stimuli after it being devaluated even though it generally was positive.
